# Measuring the impact of caregiving on informal carers: a construct validation study of the CarerQol instrument

**DOI:** 10.1186/1477-7525-11-173

**Published:** 2013-10-21

**Authors:** Renske J Hoefman, Job van Exel, Werner BF Brouwer

**Affiliations:** 1 Erasmus University Rotterdam, Institute of Health Policy and Management, P.O. Box 1738, 3000 DR Rotterdam, The Netherlands

**Keywords:** Informal care, CarerQol instrument, Impact of caregiving, Construct validation, Economic evaluations

## Abstract

**Background:**

Informal caregivers provide a significant part of the total care needed by ill or disabled persons. Although informal care is often the preferred option of those who provide and those who receive informal care, caring can nevertheless be very straining. This study investigates construct validation of an instrument of the impact of caregiving, the CarerQol.

**Methods:**

Data was collected among adult caregivers (n = 1,244) selected from the general population using an online questionnaire in October 2010, in the Netherlands. The CarerQol measures and values the impact of informal care. The CarerQol measures subjective burden (CarerQol-7D) and well-being (CarerQol-VAS). Construct validation comprised clinical, convergent and discriminative validity tests.

**Results:**

Clinical validity was supported by statistically significant associations of CarerQol-VAS and caregivers’ health, income and employment status, care recipients’ health, and the relationship between caregiver and care recipient. Convergent validity was supported by positive associations of CarerQol-VAS with the two positive CarerQol-7D dimensions (fulfillment and support) and negative associations with the five negative CarerQol-7D dimensions (relational problems, mental health problems, problems combining daily activities, financial problems and physical health problems). Moreover, CarerQol-VAS was negatively associated with other instruments measuring caregiving burden.

**Conclusions:**

Construct validity tests in a large, heterogeneous sample of caregivers show that the CarerQol validly measures the impact of caregiving. The CarerQol can be used in informal care research and economic evaluations of health care interventions. Hence, its use can facilitate informed decision making in health care.

## Background

The attention for informal care appears to be increasing, given the inherent and increasingly noticed scarcity of formal health care resources in many Western countries [1]. Informal care is an important part of total care, especially in the context of chronic illness and frailty due to ageing, and is often provided voluntarily by family, friends or acquaintances. Informal care may reduce the pressure on the capacity and budget of formal health care [2-4]. Moreover, it may be preferred by both patient and informal caregiver over formal care [5].

Notwithstanding the fact that providing informal care can be rewarding [5,6], caring can have considerable negative effects on the health and general well-being of informal caregivers [7-13]. Therefore, the impact of providing informal care on carers should be recognised by policy makers when making decisions concerning the structure and provision of health care services. Moreover, information on the impact of informal care is valuable input for policy decisions regarding arrangements facilitating and supporting informal caregiving in health care.

Economic evaluations aim to support optimal allocation of scarce health care resources. Although inclusion of informal care in economic evaluations is highly desirable [14-17], at present informal care commonly is ignored in economic evaluations. Thus, policymakers remain ignorant of the impact of interventions in health care on informal caregivers and risk making non-optimal decisions. Moreover, in the few instances that informal care is included in economic evaluations, the comparability of results is hampered by differences in measuring and valuing informal care [18-20]. This is, for example, reflected in different approaches to measure and value caregivers’ time input [19-22], health [11,23], and well-being [19,24].

Common approaches to value informal care, such as the willingness to pay method or the proxy good method, typically provide limited information regarding the underlying informal care situation and its potentially diverse impact [20,25-27]. Subjective burden measures for informal care focus more on this latter issue. Several generic and disease-specific subjective burden instruments are available describing the negative impacts of caring, such as problems experienced with mental health, physical health, or social and financial aspects [21,28-32]. Some instruments aim to capture the positive impacts of caring as well [25,29,33-35].

While many of these subjective burden instruments provide a detailed description of caregiving burden, they do not value the impact of caregiving in economic terms, making them unsuitable for economic evaluations. At this time, only two instruments combine an economic valuation of informal care with the informational density of burden instruments: The Caregiver Experience Scale (CES) [35,36] and Care-related Quality of Life instrument (CarerQol) [34]. Both instruments describe the care situation in terms of the negative and positive impact of caregiving, and value the overall impact of informal care. The CarerQol instrument values this impact in two ways: general well-being and care-related quality of life. The (latter) utility scores for the CarerQol are based on preference information from the general public in the Netherlands [37]. The CES instrument values the impact of caregiving with care-related quality of life scores, based on preference information from caregivers of elderly persons in the UK for the CES [35,36].

When patient interventions are compared in economic evaluations, the CES or CarerQol can be used as an additional source of information in cost-effectiveness analyses using conventional outcome measures, such as patient Quality Adjusted Life Years (QALYs), or as one of the principal outcome measures in cost-consequence or multi-criteria analyses. Furthermore, cost-effectiveness analyses of interventions or support programmes targeted directly at informal caregivers can apply the CES or CarerQol as main outcome.

**Figure 1:**
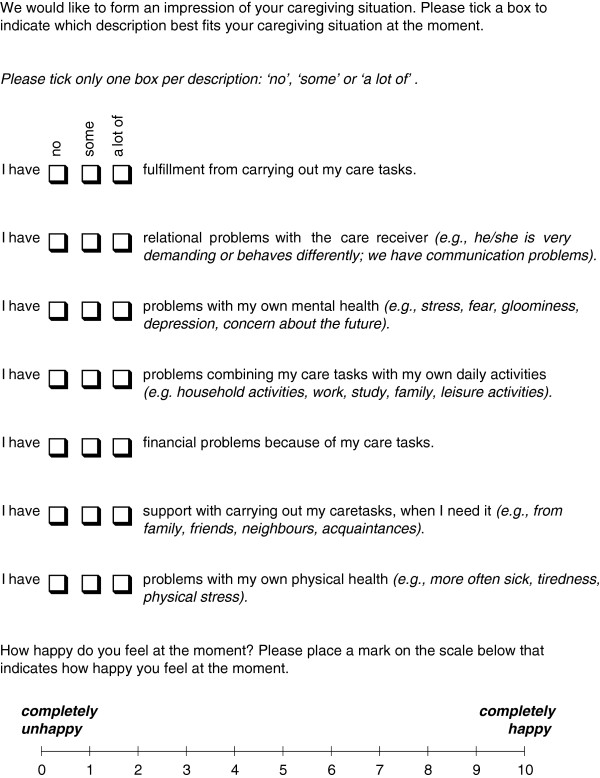
**CarerQol instrument**[37]**.**

The focus in this paper is on the CarerQol (see Figure 1). This instrument was developed in 2006, in a similar way as the EuroQol instrument [38], and it has been applied in several studies since [26,39-45].

Four validation studies of the CarerQol have been conducted previously. Brouwer et al. [34] & Hoefman et al. [46] performed tests of the feasibility of the CarerQol instrument. Construct validity of the CarerQol instrument was studied in different caregiver samples: two heterogeneous groups of caregivers that were members of regional caregiver support centres in the Netherlands (n = 175 in Brouwer et al. [34] and n = 230 in Hoefman et al. [47]), caregivers of persons permanently living in or receiving day care from a nursing home in the Netherlands (n = 108) [46] and a sample of children with craniofacial malformations living in the US (n = 65) [44]. Test-retest reliability of the CarerQol was also investigated in the sample of caregivers of nursing home care patients [46]. These various tests of the psychometric properties of the CarerQol showed favourable results concerning its feasibility [34,46], construct validity [34,44,46,47], and test-retest reliability [46].

The results of these four studies require further confirmation for several reasons. First, the validation studies so far used caregiver samples that were relatively small: the number of respondents ranged between 65 and 230. Second, these samples were either overrepresented by relatively strained caregivers [34,47] or by caregivers in a specific informal care situation (e.g., caring for young disabled children [44] or institutionalized elderly [46]). Third, the range of tests used for construct validation was limited. Most tests concerned whole sample analyses and did not investigate possible heterogeneity among caregivers (because of study sample size). Moreover, few other subjective burden instruments were available from these studies to compare the CarerQol with (e.g. [44,46]).

The study presented in this paper aimed to overcome most of these shortcomings by using a much larger, heterogeneous sample of informal caregivers (n = 1,244), representing a broad range of informal care situations and levels of caregiving burden. This sample size also allows for the construct validation of the CarerQol to be tested in a more elaborate way, which is important given the lack of a gold standard for the impact of caregiving. These tests will be conducted by comparing the performance of the CarerQol with a number of other subjective burden instruments and a range of subgroup analyses comparing between groups of carers characterised by differences in caregiving strain, health and socio-economic characteristics.

This study was specifically designed to validate the CarerQol and to demonstrate its ability to assess the overall impact of caregiving in diverse types of caregiving situations. The availability of a validated instrument to measure and value the impact of caregiving will support its application in informal care research. Moreover, it facilitates the inclusion of informal care impacts in economic evaluations of diverse patients and caregiver interventions, and better evidence-based decision making in health care.

## Methods

### Data

Data was gathered using an online questionnaire in October 2010. A sample that was representative of the adult Dutch population in terms of age and gender was recruited from a large online panel. From this sample informal caregivers were selected. This was done by asking whether respondents (1) provided care or support, on a voluntarily basis, to a family member, friend or acquaintance who needed help due to physical or mental health problems or problems due to aging, and (2) for how long they have been lending this care. These selection questions ensured that data would only be gathered among respondents who had been lending informal care for more than two weeks. The questionnaire on informal care was completed by 1,288 respondents of which 44 were dropped from the final sample for analysis. Main reasons were an unrealistically short completion time (i.e. respondents rushing through the questionnaire) or the fact that the answers indicated the respondent was not an informal caregiver after all. The latter was typically the case when the answers indicated a respondent worked for a voluntary organization or provided zero hours of care per week. This left 1,244 questionnaires in the final sample.

### Questionnaire

The questionnaire was based on the iMTA Valuation of Informal Care Questionnaire (iVICQ) [37] and included questions on the impact of caregiving as well as characteristics of the caregiver, care recipient and the care situation.

The impact of caregiving was measured with the CarerQol instrument, ASsessment of the Informal care situation Scale (ASIS), the Self-Rated Burden scale (SRB), the Process Utility measure (PU), the Caregiver Strain Index (CSI) and Perseverance time (Pt).

The CarerQol measures well-being (CarerQol-VAS) and subjective burden (CarerQol-7D). Well-being is measured in terms of happiness using a visual analogue scale (VAS) with endpoints 'completely unhappy’ (0) and 'completely happy’ (10) (CarerQol-VAS) [34]. Subjective burden is measured on seven dimensions (CarerQol-7D): fulfillment (positive dimension), relational problems (negative dimension), mental health (negative), daily activities problems (negative), physical health (negative) and support (positive). Respondents describe their caregiving situation by selecting one of three possible responses on each dimension: (i) no, (ii) some, and (iii) a lot. The combination of dimensions and answering categories discerns a total of 2187 (= 3^7^) caregiving situations. Tariffs are available to compute a weighted sum score for the CarerQol-7D, which represents informal care situation utilities ranging from 0 (worst informal caregiving situation) to 100 (best informal caregiving situation) [37,48]. Like common health-related quality of life measures for patients [49,50], the tariffs for the CarerQol-7D were based on preferences of the general public for different care situations as described with the seven dimensions (and three levels) of the instrument.

The ASIS measures the desirability of the caregiving situation with a horizontal VAS ranging from (0) the 'worst imaginable caregiving situation’ to (10) the 'best imaginable caregiving situation’ [46]. The SRB measures the subjective burden of informal care with a horizontal VAS ranging from (0) 'not straining at all’ to (10) 'much too straining’ [25,29]. PU measures the value attached to the process of caregiving by comparing caregivers’ current well-being with their well-being in a hypothetical situation that all caregiving tasks would be taken over by someone selected by the caregiver and care recipient, free of costs [5].

The CSI [31] measures the strain of caregiving by asking caregivers’ experiences in 13 common problem areas, leading to a non-weighted sum score ranging from 0 to 13. Higher scores indicate higher burden, and caregivers are considered to experience substantial strain when their score is 7 or higher [31]. In addition, we combined the CSI with the five positive aspects of caregiving that caregivers may experience, forming the CSI + as proposed by Al-Janabi et al. (2010) [33]. Finally, Pt indirectly measures the burden of caregiving by asking caregivers how long they expect to be able to continue performing their current informal care tasks if the care situation remains stable, with pre-defined answer categories ranging from less than two weeks to more than two years [51].

Informal caregiver characteristics collected were age, gender, highest attained educational level, performing paid work, household income, health (using EQ-5D [38]) and having a partner. Care recipient characteristics included age, gender, health (using EQ-5D [38]), level of independence (using KATZ scale [52,53]), need for 24/7 surveillance and type of health problem (defined as a temporary or chronic condition). The care situation was described by the relationship and co-residence of care recipient and caregiver, the number of years, days per week and hours per day caregiving, use of home care, day care, or other institutional care, being on waiting list for day or nursing care, and support from other informal caregivers.

### Statistical analyses

Descriptive statistics in percentages and means were calculated of the characteristics of caregivers, care recipients and informal care situations and of the instruments measuring the impact of informal care.

Three types of construct validation were studied: clinical, convergent and discriminative validity. Clinical validity concerns the extent to which the measure relates to variables, such as important background characteristics [54]. Convergent validity is assessed by considering whether a construct of a measure resembles that of other instruments with the same subject of measurement [54]. Discriminative validity tests can be used to study whether 'extreme’ groups of respondents score differently on an instrument [54].

#### *Clinical validity*

Clinical validity was investigated by studying the association between CarerQol-VAS scores and background characteristics of the caregiver, care recipient and informal care situation with one-way ANOVA tests and Spearman’s correlation coefficients. Multivariate associations were analysed with ordinary least squares regression (OLS) of CarerQol-VAS scores and caregiver, care recipient and informal care situation characteristics, correcting for subjective burden (CarerQol-7D). Statistically insignificant variables were excluded from the model. We used a less restrictive p-value of 0.2 for this, to avoid excluding variables that did explain variance in CarerQol-VAS scores [55]. Reference values for categorical variables were those with the highest CarerQol-VAS score. To avoid the problem of too few respondents per category, we merged categories of variables when one of the categories represented 10% or less of the data. Missing values for duration of caregiving (missing value in 7 cases) and income (missing value in 342 cases) were supplemented in the multivariate analysis using the multiple imputation by chained equations (MICE) command in Stata [56,57].

#### *Convergent validity*

Convergent validity of the CarerQol was studied by (i) the association between the two parts of this instrument, and by (ii) the association between the CarerQol and other instruments measuring the impact of caregiving included in the questionnaire: ASIS, SRB, PU, CSI and Pt.

Spearman’s correlation coefficients were used to study bivariate associations of CarerQol-VAS and CarerQol-7D. Multivariate associations were studied using OLS. Subgroup analyses of the multivariate associations of CarerQol-VAS and CarerQol-7D were performed based on a low or high score on the ASIS, SRB, PU, CSI or Pt. Background characteristics that appeared important in explaining CarerQol-VAS (in clinical validity) were also used to construct subgroups.

Furthermore, associations of CarerQol-VAS and CarerQol-7D dimensions with ASIS, SRB, PU, CSI and Pt were inspected using Spearman’s correlation coefficients. Correlations <0.1 were considered as trivial; 0.1–0.3 as small; 0.3–0.5 as moderate; 0.5–0.7 as high; 0.7–0.9 as very high; >0.9 as nearly perfect [58].

#### *Discriminative validity*

Discriminative validity of the CarerQol was investigated by contrasting extreme subgroups [54]. Specifically, we studied differences in mean scores on the instruments measuring the impact of caregiving among respondents scoring a 'no’ or 'a lot’ on CarerQol-7D dimensions with Student’s t-tests.

All statistical analyses were performed with STATA 11.0 (Statistics/Data Analysis).

### Ethics

No ethics approval was required for this study. We collected data from an online panel. People subscribing to this panel are informed about privacy and data use issues. After deciding to subscribe, people regularly receive invitations to participate in all sorts of online surveys and they are free to accept any invitation they receive. In the case of this study, people received information about the purpose of the study and the organization conducting it (our institute), the type of questions and the estimated completion time. People accepting the invitation were directed to the online questionnaire. After starting completion, they were free to terminate their participation at any point. People submitting their data at the end of the questionnaire were assumed to approve of the content of the questionnaire and their response, and to give consent for use of their response for the purpose of our study, as stated in the invitation. People received a small incentive for completing a questionnaire: after submitting their data, they were offered the opportunity to donate a small sum, depending on the length of the questionnaire, to a charity of their choice. The data we received from the survey sampling organization was anonymous.

## Results

### Study sample

Table 1 presents informal caregiver, care recipient and care situation characteristics. The mean age of caregivers was 47 years. A slight majority of them were female. Somewhat more than fifty per cent had a paid job. Care recipients were on average 63 years old and two thirds of them were female. Their average EQ-5D score was 0.5. Most caregivers lent care to their parents (in-law). On average, caregivers had been providing care for 5 years and spent 18 hours per week on care.

**Table 1:** **Sample characteristics and association with CarerQol-VAS; n = 1,244**

			**%**	**CarerQol-VAS mean**	**P-value**^**a**^	**Spearman’s rho**	**Standardised coefficients**^**b**^
Caregiver	Age	<47.1 years	47.1	7.2	0.20	-0.04	-0.06
		≥ 47.1 years		7.1			
	Gender	Female	58.3	7.0	0.02	-	-
		Male	41.7	7.3			
	Educational level	Low	14.6	7.2	0.83	-	-
		Middle	55.9	7.1			
		High	29.6	7.1			
	Paid work	Yes, full-time	26.7	7.4	0.00	-	ref.
		Yes, part-time	27.0	7.2			-0.07^*^
		No	46.3	6.9			-0.06
	Income (1-15)^c^					0.11^***^	
	Income^d^	Low	31.7	7.0	0.04	-	-0.10^*^
		Middle	24.0	7.1			-0.07
		High	16.9	7.4			ref.
		Missing	27.5	7.1			
	EQ-5D score	<0.8	0.84	6.4	0.00	0.35^***^	0.08^*^
		≥0.8		7.5			
	Having a partner	Yes	68.9	7.2	0.09	-	-
		No	31.1	7.0			
Care recipient	Age	< 63.6 years	63.6	7.0	0.01	0.06^*^	0.06
		≥ 63.6 years		7.2			
	Gender	Female	66.2	7.2	0.04	-	-
		Male	33.8	7.0			
	EQ-5D score	<0.5	0.5	6.8	0.00	0.21^***^	0.09^**^
		≥0.5		7.4			
	Level of independence (1-6)	<4.3	4.3	7.2	0.03	0.06^*^	-
		≥4.3		7.0			
	Surveillance needed 24/7	Yes	11.9	6.9	0.10	-	-
		No	88.1	7.2			
	Type of health problem	Temporary condition	10.3	7.5	0.01	-	-
		Chronic condition	89.7	7.1			
Care situation	Relationship with care recipient	Partner	15.3	7.0	0.02	-	0.02
		Parent (-in-law)	41.6	7.0			-0.09^*^
		Other family	24.4	7.2			-0.01
		Friend / acquaintance	18.8	7.4			ref.
	Care recipient shares household	Yes	31.0	7.0	0.02		-
		No	69.1	7.2			
	Total years care	<5.1 years	5.1	7.2	0.05^e^	-0.05	-
		≥5.1 years		7.0			
	Days p/wk	<4.1 days	4.1	7.2	0.12	-0.04	-
		≥ 4.1days		7.1			
	Hours p/wk	<18.4 hours	18.4	7.2	0.10	-0.06^*^	-
		≥18.4 hours		7.0			
	Professional care^e^	Yes	49.6	7.0	0.00	-	-
		No	50.3	7.2			
	Day care^e^	Yes	8.7	6.9	0.22	-	-
		No	91.3	7.1			
	Other informal caregivers	Yes	31.4	7.2	0.09	-	-
		No	68.7	7.1			
	Institutionalization	Yes	24.8	7.1	0.83	-	-
		No	75.2	7.1			
	Waiting list^e^	Yes	11.9	7.1	0.89	-	-
		No	88.1	7.1			

Note: *p < 0.05, **p < 0.01, ***p <0.001; ^a^oneway anova test; ^b^from multivariate regression analysis corrected for CarerQol-7D; ^c^continuous variable, missings not included; ^d^categorical variable; ^e^caregivers of non-institutionalized care recipients only, n = 1062.

### Instruments

The mean CarerQol-7D score was 79.1. The majority of caregivers derived a lot of fulfillment from caregiving. Problems most often encountered were physical health problems and problems with daily activities. Around one third had relational, mental health or financial problems. Most of the caregivers experienced only mild problems. Just over one fourth did not receive support with caregiving when needed. The mean CarerQol-VAS score was 7.1.

The mean ASIS score was 6.7 and the mean SRB score was 4.1. Overall, PU was positive with a mean of 1.6 (implying that the happiness of these caregivers would drop with 1.6 point when handing over all care tasks) and two thirds of carers indicated to have a positive PU score. The mean CSI score was 4.8 and 29 per cent experienced 'substantial strain’. On average, caregivers expected to be able to persevere with their care task for two years or more (Table 2).

**Table 2:** Descriptive statistics of CarerQol, ASsessment of Informal care Situation (ASIS), Self-Rated Burden (SRB), Process Utility (PU), Caregiver Strain Index (CSI) and Perseverance time (Pt), n = 1,244

		**%**	**mean (SD)**
CarerQol-7D (0-100)			79.1 (18.6)
▪ Fulfillment	- no	6.7	
	- some	30.9	
	- a lot	62.5	
▪ Relational problems	- no	64.7	
	- some	28.9	
	- a lot	6.4	
▪ Mental health problems	- no	58.2	
	- some	31.2	
	- a lot	10.5	
▪ Problems combining daily activities	- no	50.3	
	- some	39.1	
	- a lot	10.6	
▪ Financial problems	- no	67.9	
	- some	23.9	
	- a lot	8.3	
▪ Support	- no	27.0	
	- some	47.2	
	- a lot	25.8	
▪ Physical health problems	- no	49.4	
	- some	36.4	
	- a lot	14.2	
CarerQol-VAS (0-10)		7.1 (1.6)	
ASIS (0-10)		6.7 (1.9)	
SRB (0-10)		4.1 (2.5)	
PU	score		1.6 (2.8)
	- positive	66.5	
	- neutral	8.8	
	- negative	24.8	
CSI	score		4.8 (3.2)
	- substantial burden (>6)	29.1	
Pt	in months		23.4 (0.3)
	- less than one week	2.7	
	- less than one month	3.1	
	- less than six months	7.5	
	- more than six months, but less than a year	8.4	
	- more than one year, but less than two years	9.8	
	- more than two years	68.6	

### Clinical validity CarerQol-VAS

Bivariate analyses of CarerQol-VAS scores and background characteristics (Table 1) showed that CarerQol-VAS was higher among male caregivers, caregivers with a paid job, in particular a full-time position, caregivers with high income, and caregivers in relatively good health. CarerQol-VAS seemed higher among caregivers lending care to older, healthier or female care recipients. Care situation characteristics that had a bivariate association with CarerQol-VAS were duration and intensity of caregiving, relationship between caregiver and care recipient and sharing a household. Multivariate analysis showed that caregivers’ and care recipients’ health status were positively associated, and having a part-time job, a low income or giving care to parents (in-law) were negatively associated with CarerQol-VAS.

### Convergent validity

#### *CarerQol-VAS & CarerQol-7D*

Table 3 shows that CarerQol-VAS was positively associated with the positive dimensions of the CarerQol-7D fulfillment and support, and negatively with the negative dimensions of CarerQol-7D. Multivariate association of CarerQol-VAS and CarerQol-7D (Table 4) confirmed that CarerQol-VAS score were higher among caregivers that experienced fulfillment and received support and when problems were absent. However, relational and financial problems were insignificant in this model.

**Table 3:** Spearman’s correlation coefficients of the CarerQol instrument and ASsessment of Informal care Situation (ASIS), Self-Rated Burden (SRB), Process Utility (PU), Caregiver Strain Index (CSI), and Perseverance time (Pt; in months), n = 1,244

		**CarerQol-VAS**	**ASIS**	**SRB**	**PU**	**CSI**	**Pt**
CarerQol-VAS		-	0.31	-0.33	0.52	-0.40	0.22
CarerQol-7D	- Fulfillment	0.24	0.24	-0.30	0.31	-0.25	0.29
	- Relational problems	-0.19	-0.25	0.35	-0.28	0.38	-0.26
	- Mental health problems	-0.36	-0.24	0.39	-0.32	0.47	-0.30
	- Problems combining daily activities	-0.27	-0.25	0.47	-0.30	0.52	-0.36
	- Financial problems	-0.24	-0.24	0.30	-0.22	0.42	-0.26
	- Support	0.14	0.13	-0.10	0.09	-0.12	0.02
	- Physical health problems	-0.35	-0.22	0.42	-0.27	0.48	-0.25

Note: All Spearman’s correlation coefficients are statistically significant at a 99% C.I., except the correlation coefficient of 'support’ and PU which is significant at a 95% C.I, and 'support’ and Pt which is ns.

**Table 4:** Multivariate regression analysis of CarerQol-VAS, standardized coefficients

**CarerQol-7D**		**Whole sample**	**Subgroup analyses**															
			**ASIS**		**SRB**		**PU**		**CSI**		**Pt**		**EQ-5D score**		**EQ-5D score**		**income**	
													**caregiver**		**care recipient**			
			**low**	**high**	**low**	**high**	**low**	**high**	**low**	**high**	**low**	**high**	**low**	**high**	**low**	**high**	**low**	**high**
			**(n = 484)**	**(n = 421)**	**(n = 465)**	**(n = 413)**	**(n = 417)**	**(n = 409)**	**(n = 508)**	**(n = 362)**	**(n = 391)**	**(n = 853)**	**(n = 457)**	**(n = 787)**	**(n = 430)**	**(n = 439)**	**(n = 394)**	**(n = 210)**
Fulfillment	- no	-0.12^***^	0.02	-0.12^*^	-0.20^***^	0.11^*^	-0.13^**^	0.01	-0.19^***^	-0.13^**^	0.06	-0.18^***^	-0.04	-0.20^***^	-0.06	-0.28^***^	-0.10^*^	-0.17^*^
	- some	^a^	-0.12^**^	^a^	^a^	-0.10^*^	^a^	^a^	^a^	^a^	-0.08	^a^	^a^	^a^	^a^	^a^	^a^	^a^
Relational problems	- some	-0.01	-0.06	0.02	-0.01	-0.06	0.04	-0.02	0.04	-0.05	-0.01	-0.02	-0.02	-0.02	-0.01	0.01	0.03	0.08
	- a lot	^b^	0.00	^b^	^b^	0.00	0.04	^b^	^b^	-0.03	-0.03	^b^	^b^	^b^	^b^	^b^	^b^	^b^
Mental health problems	- some	-0.14^***^	-0.13^**^	-0.23^***^	-0.18^**^	-0.09	-0.18^**^	-0.08	-0.15^**^	-0.05	-0.10	-0.21^***^	-0.19^***^	-0.06	-0.13^*^	-0.22^***^	-0.21^***^	-0.06
	- a lot	-0.21^***^	-0.28^***^	^b^	^b^	-0.36^***^	-0.38^***^	^b^	^b^	-0.22^**^	-0.21^**^	^b^	-0.36^***^	^b^	-0.32^***^	^b^	-0.27^***^	-0.24^**^
Problems with daily activities	- some	-0.04	-0.01	-0.06	-0.03	-0.02	0.02	-0.05	-0.01	-0.13^**^	-0.06	-0.02	-0.01	-0.06	-0.02	-0.09^c^	-0.07	-0.09
	- a lot	-0.08^**^	-0.17^**^	-0.05	^b^	-0.08	-0.03	^b^	^b^	-0.27^***^	-0.12	^b^	-0.15^*^	^b^	0.00	^b^	^b^	-0.03
Financial problems	- some	-0.03	0.03	-0.06	-0.01	0.01	-0.02	-0.05	-0.09	0.00	0.01	-0.07^*^	-0.01	-0.08^*^	-0.03	-0.03	-0.12^*^	0.07
	- a lot	^b^	0.03	^b^	^b^	0.05	-0.00	^b^	^b^	0.00	0.01	^b^	-0.01	^b^	0.01	^b^	-0.07	^b^
Support	- no	-0.11^***^	-0.15^**^	-0.05	-0.06	-0.14^*^	-0.15^**^	-0.05	-0.05	-0.15^*^	-0.20^**^	-0.06	-0.12^*^	-0.10^*^	-0.12^*^	-0.10^*^	-0.09	-0.03
	- some	-0.07^**^	-0.06	-0.10	-0.04	-0.07	-0.11	-0.03	-0.03	-0.09	-0.13^*^	-0.03	-0.10^*^	-0.05	-0.05	-0.07	-0.09	-0.06
Physical health problems	- some	-0.12^***^	-0.13^*^	-0.11^c^	-0.14^**^	-0.14^*^	-0.07	-0.17^**^	-0.14^**^	-0.17^*^	-0.10	-0.14^**^	-0.05	-0.13^**^	-0.11	-0.08	-0.09	-0.18^*^
	- a lot	-0.13^***^	-0.15^**^	^b^	^b^	-0.22^***^	-0.13^*^	^b^	^b^	-0.18^*^	-0.19^**^	^b^	-0.04	^b^	-0.13^*^	^b^	-0.04	-0.13
Constant (coefficient)		8.13	7.96	8.34	8.27	8.04	7.60	8.33	8.15	8.34	8.04	8.11	7.78	8.14	7.84	8.41	7.39	8.31
Adjusted R^2^		0.21	0.25	0.16	0.14	0.25	0.23	0.07	0.13	0.21	0.17	0.18	0.17	0.14	0.16	0.25	0.23	0.24

Note: *p < 0.05, **p < 0.01, ***p <0.001, Reference category is 'a lot’: Fulfillment, Support, reference category is 'no’: Relational problems, Mental health problems, Problems with daily activities, Financial problems, Physical health problems, ^a^categories are 'no or some’ versus 'a lot’, ^b^categories are 'some or a lot’ versus 'no’, ^c^p-value is 0.05, ^d^p-value is 0.08.

Subgroup analyses (Table 4) showed that problems with daily activities and support were associated with CarerQol-VAS among relatively burdened caregivers. In addition, problems with daily activities were associated with CarerQol-VAS among caregivers in relatively bad health, and fulfillment among those in relatively good health. Among carers of care recipients with a relatively good health status, fulfillment was associated with CarerQol-VAS, and problems with physical health were associated with CarerQol-VAS in the subgroup of care recipients with bad health. Furthermore, financial problems were associated with CarerQol-VAS among caregivers indicating a long perseverance time, carers of their parents and carers with low income, while having physical health problems was associated with CarerQol-VAS among high income carers.

#### *CarerQol & ASIS, SRB, PU, CSI, Pt*

Table 3 presents the Spearman’s correlation coefficients of the CarerQol and the five other instruments. CarerQol-VAS had a positive association with ASIS, PU and Pt, and a negative association with SRB and CSI. CarerQol-7D’s positive dimensions had a statistically significant positive association with ASIS, PU and Pt, and a negative one with SRB and CSI. CarerQol-7D support and Pt were not significantly associated. The negative CarerQol-7D dimensions were all negatively associated with ASIS, PU and Pt, and positively with SRB and CSI.

Concerning convergent validity of single CarerQol-7D dimensions (results not presented in a table), the CarerQol-7D item fulfillment had a positive association with CSI 'happy to care’ (correlation coefficient (cc) 0.27) and 'care is important’ (cc 0.23). CarerQol-7D dimension relational problems was associated with 'recipient appreciates care’, 'emotional adjustments’, 'behaviour upsetting’ and 'recipient change upsetting’ (absolute range cc 0.25-0.43). CarerQol-7D dimension mental health was associated with 'emotional adjustments’, 'behaviour upsetting’, 'recipient change upsetting’, 'sleep disturbed’, 'inconvenient’ and 'feel completely overwhelmed’ (absolute range cc 0.18-0.40). CarerQol-7D dimension daily activities was associated with 'confining’, 'enough time for self’,’ family adjustments’, 'changes in personal plans’ and 'work adjustments’ (absolute range cc 0.29-0.47). CarerQol-7D’s financial problems was positively associated with CSI 'work adjustments’ (cc 0.30) and 'financial strain’ (cc 0.57). CarerQol-7D physical health problems was associated with 'inconvenient’, 'feel completely overwhelmed’, 'physical strain’, 'handle the care fine’ and 'sleep disturbed’ (absolute range cc 0.24-0.40).

### Discriminative validity

Table 5 shows the mean values of CarerQol-VAS, ASIS, SRB, PU, CSI and Pt per extreme level of CarerQol-7D. Respondents with a lot of fulfillment or support, or no problems on the negative dimensions, had statistically significant higher mean CarerQol-VAS, ASIS, and PU scores, and lower SRB and CSI scores than respondents scoring the other extreme level. The same result applied to Pt, however there was no statistically significant difference in perseverance time among caregivers receiving no or a lot of support. All respondents with a lot of problems on negative CarerQol-7D dimensions experienced 'substantial strain’ on the CSI. Caregivers with no fulfillment or no support had a mean CSI value lower than the CSI cut-off point for substantial strain.

**Table 5:** Mean values of CarerQol-VAS, ASsessment of Informal care Situation (ASIS), Self-Rated Burden (SRB), Process Utility (PU), Caregiver Strain Index (CSI) and Perseverance time (Pt; in months) per 'extreme level’ of CarerQol-7D; n = 1,244

**CarerQol-7D**		**CarerQol-VAS**	**ASIS**	**SRB**	**PU**	**CSI**	**Pt**
Fulfillment	- no (n = 83)	6.9	5.9	5.9	0.6	6.4	11.7
	- a lot (n = 777)	7.4	7.0	3.6	2.3	4.2	25.6
Relational problems	- no (n = 805)	7.4	7.0	3.5	2.2	3.9	25.4
	- a lot (n = 80)	6.6	5.6	6.0	0.0	7.4	18.4
Mental health problems	- no (n = 724)	7.6	7.1	3.3	2.3	3.5	25.8
	- a lot (n = 131)	5.7	5.7	6.1	-0.4	7.7	16.5
Problems combining daily activities	- no (n = 626)	7.5	7.2	3.0	2.4	3.2	26.8
	- a lot (n = 132)	5.9	5.8	6.4	-0.2	8.2	14.5
Financial problems	- no (n = 844)	7.4	7.0	3.6	2.0	3.8	25.2
	- a lot (n = 103)	6.5	5.7	5.7	0.7	7.7	16.5
Support	- no (n = 336)	6.9	6.5	4.3	1.5	5.2	23.3
	- a lot (n = 321)	7.5	7.2	3.6	2.1	4.1	23.7
Physical health problems	- no (n = 614)	7.7	7.1	3.1	2.3	3.3	25.6
	- a lot (n = 177)	6.1	5.8	6.0	0.2	7.2	17.3

Note: All differences in means are statistically significant at a 99% C.I., except Support & PU: significant at C.I. 95%, Support & Pt is ns.

## Discussion

This study investigated whether the CarerQol validly assessed the overall impact of caregiving in a large, heterogeneous sample of caregivers from the Netherlands. Results of clinical, convergent and discriminative validity tests confirmed the favourable results from previous studies. Both the subjective burden (CarerQol-7D) and the well-being (CarerQol-VAS) component of the CarerQol were significantly associated in the expected direction with other measures of the impact of caring. Additionally, well-being was related to important caregiver, care recipient and care situation characteristics, in expected directions. Hence, this study adds to previous evidence suggesting that the CarerQol may be a useful measure of the impact of caregiving in research in a wide variety of informal care contexts. Moreover, it facilitates inclusion of informal care in economic evaluations of health care interventions.

The CarerQol instrument measures care-related quality of life of caregivers. This concept is broader than the generally used outcome measure in economic evaluations, as for instance health-related quality of life in cost-utility analyses. Therefore, the results of the CarerQol cannot be easily combined with patient outcomes cost-effectiveness or -utility analyses. Nevertheless, as stated in the Introduction, the results of the CarerQol can be presented alongside the results of an economic evaluation and so inform decision makers more completely about the total impact of an intervention on society. The CarerQol can also be used with other types of economic evaluation, such as multi-criteria analyses, which allow considering multiple outcome measures. Finally, the CarerQol can very well serve as the main outcome measure in economic evaluations of programmes for caregivers (e.g., support programmes, respite care).

Before we discuss the implications of our results in more detail, it is important to note some limitations of our study. First, although we used a representative sample of the adult Dutch population in terms of age and gender, our sample may be somewhat selective due to the use of an online panel. Internet was considered to be a suitable medium to gather data, because more than 90 per cent of the Dutch population uses internet. In addition, while elderly may be a typical group expected to be underrepresented in internet surveys, it needs noting that in recent years elderly became more active on the internet with six out of ten elderly of 65 to 75 years currently being internet users [59]. Furthermore, selection bias could have occurred in using an online panel to select caregivers. We did not have a priori reasons to suspect that caregivers in general would be less likely to subscribe to online panels. This may be the case for caregivers experiencing high levels of strain, but this group is generally difficult to approach and less likely to participate in any type of survey. Hence, we expect that the subgroup of caregivers experiencing severe strain may be underrepresented in our sample, but not more or less than in other studies among caregivers. On the other hand, in previous validation studies [34,44,46,47] caregivers were recruited via organisations providing information and support services for caregivers. It is likely that people who come to such organisations to ask for assistance see themselves as caregivers and concern a selection of caregivers experiencing relatively higher strain in their caregiving situation. Through the online panel and the selection questions used in this study it is likely that we have reached persons lending care who would normally not define themselves as caregivers, for instance because their burden is low, and therefore would not be represented in these previous studies. All in all, we expect our sample to be more representative of the caregiver population, in particular at the low burden end.

Second, in our multivariate models, we used the multiple imputation method (MI) to handle missing values. An assumption of this method is that these values are, at least, missing at random [56,60]. While income is a classic example of missing not at random [61], we nevertheless considered MI as the best alternative to other strategies, such as excluding respondents with missing values from our model, or mean imputation. In addition, income correlated with other background characteristics in our data, such as gender of the caregiver, which gives some support to our imputation method.

Third, validation is an on-going process, and therefore, testing psychometric properties among caregivers in other settings, such as caregivers in other countries than the Netherlands, as for instance recently in the US [44], remains desirable. In addition, other psychometric properties of the CarerQol could be further tested, such as reliability [46] and sensitivity to change.

The overall well-being in this sample was relatively high compared to caregiver samples of previous validation studies. Possibly this is due to the recruitment procedure in this study that may have attracted significantly more caregivers in low burden care situations, as discussed before. Higher well-being scores were found among caregivers with positive care experiences in terms of fulfillment from caregiving and assistance from others in lending care, which many caregivers reported to have. Furthermore, as previous CarerQol validation and informal care studies underlined, well-being of caregivers was positively related to health of both the caregiver and care recipient [23,34,46,47,62,63]. Moreover, as other studies on informal care also suggest (e.g. [23,62], well-being of carers was positively influenced by more general aspects of life, not necessarily (directly) related to caregiving [64], such as having a full-time paid work position or a high income. Besides these positive influences, caregivers also experienced negative consequences of lending care. Important to note here, is that we used cross-sectional data and hence were not able to determine the causality of the established associations. Caregivers often faced problems with their health and also reported difficulties combining care with other daily activities. Having these problems, negatively affected their well-being. Further, especially those caring for their parents (in-law) reported lower happiness scores.

Our study clearly indicates that the diverse problems associated with informal care are not equally important for all caregivers. For example, financial problems were negatively associated with happiness of caregivers with a relatively low income particularly. In addition, mental health problems and problems with daily activities were associated with well-being among caregivers with a relatively low health status, while physical health problems and financial problems were among relatively healthy caregivers. Differences were also observed for receiving support with care tasks. The positive influence of support was especially prevalent among highly burdened caregivers.

Furthermore, a note should be made on the CarerQol-7D dimension relational problems, which showed overall satisfactory convergent and discriminative validity results, but did not impact well-being when considered in combination with other burden dimensions. This seems to contradict some previous validity results [34,46]. However, additional tests in subgroups, more closely resembling the samples used in earlier studies, confirmed that relational problems did affect well-being among carers of recipients with a persistent care need often due to chronic or age-related health problems.

This diversity in importance of burden dimensions among subgroups indicates that although some aspects of caregiving burden may not seem relevant in some group of caregivers, they may matter to caregivers in another context, as was described above for the dimension relational problems. Additionally, given that not all problems are equally important for caregivers, it is recommendable that support programmes target the problems that are relevant to specific (groups of) caregivers, such as relieving financial problems of caregivers on low income.

Convergent and discriminative validity tests using the ASIS, SRB, PU, CSI and Pt instruments, which aim to measure a similar construct as the CarerQol, showed that the CarerQol instrument performs well. That is, when the ASIS, SRB, PU, CSI and Pt reported higher caregiving burden, the CarerQol also indicated a higher negative impact of caregiving. All these associations between the CarerQol and the overall scores of the ASIS, SRB, PU, CSI and Pt were as expected and the strength of correlations was small to high. The support dimension of the CarerQol-7D showed the weakest correlation with these other measures.

The associations between single CarerQol-7D dimensions and similar individual items of the CSI confirmed our hypothesized relationships as well. The only hypothesis that was rejected was that of the CarerQol-7D dimension daily activities and the CSI item 'other demands on time’. This counterintuitive result may be explained by different content of both items, because the CSI item merely registers whether caregivers perform other activities, while the CarerQol-7D focuses on difficulties with combining these with caregiving.

Discriminative validity tests showed that the CarerQol-7D discriminated well between caregivers with low or high burden. These results were less stable for the dimension support. Although in general support is an important issue for caregivers [35], some of our validity results were less satisfactory for the support dimension of the CarerQol. This could be explained by the fact that receiving support could have both a positive and a negative effect on caregiving strain. For example, it has been shown that sharing tasks with other informal caregivers tends to decrease burden, but that cooperation with others may also increase burden in case of disagreements between caregivers [12]. Given that support from family or friends with caregiving can both relieve and intensify strain among caregivers, the overall effect of support can level out when studying a large, diverse group of caregivers. It would be interesting to investigate this in more detail in future studies using the CarerQol, for instance by adding a few supplementary questions about the amount, type and perceived quality of support.

## Conclusions

This study largely confirmed previous findings on construct validation of the CarerQol and added new, strong arguments that this instrument is a valid measure of the overall impact of informal care. Therefore, the CarerQol can be applied in both informal care research and in economic evaluations of diverse patients or caregiver interventions to reveal the important, but often hidden, impact of informal caregiving for well-informed health care policy.

## Competing interests

The authors declare that they have no competing interests.

## Authors’ contributions

RH participated in the development of the questionnaire and the data handling, carried out all analyses and drafted the manuscript. JE coordinated the questionnaire development and the data collection and handling, supervised the data analyses and provided comments to draft versions of the manuscript. WB supervised the study, participated in the development of the questionnaire, and provided comments to all analyses and draft versions of the manuscript. All authors read and approved the final manuscript.
